# Improving Health-Related Symptoms and Behaviors in Children and Adolescents Diagnosed With Diabetes by Using a Virtual Reality–Gamified Self-Care Method: Randomized Controlled Trial

**DOI:** 10.2196/81402

**Published:** 2026-06-10

**Authors:** Ruhollah Solat, Reza Pourhosein, Hadi Bahrami Ehsan, Ata Pourabbasi, Amin Hashemi, Javad Hatami

**Affiliations:** 1 Psychology Department, Tehran University, Dr. Kardan Street, Tehran, 1445983861, Iran, 98 02188250062; 2 Neuroscience Department, Tehran University, Tehran, Iran

**Keywords:** diabetes, gamification, health, self-care, virtual reality

## Abstract

**Background:**

Self-care plays an important role in improving health symptoms in patients diagnosed with chronic illnesses such as diabetes. Gamification is among the most effective methods for enhancing health monitoring by applying principles of behavioral economics and motivation.

**Objective:**

This study aimed to develop an innovative self-care system using a virtual reality (VR)–gamified intervention and to evaluate its effects on patients diagnosed with diabetes metabolic symptoms and health-related behaviors compared to other standard interventions.

**Methods:**

This study was a randomized controlled trial with a parallel-group design, conducted between December 2024 and February 2025. A total number of 78 patients diagnosed with diabetes were recruited from The Children’s Medical Center clinic in a closed, offline setting. Briefing, tutorials, and interventions were partly face-to-face. Eligible participants (n=68) were randomly assigned to the 4 groups using stratified block randomization based on age and sex, with allocation concealment ensured through a sequentially numbered process. Each group received a different type of self-care intervention for 6 weeks, which was health care provider-assisted. One group received the VR gamified intervention, while the other 3 groups received comparator interventions, including the MySugr (mySugr GmbH) monitoring app, traditional counseling, and medication only, respectively. The primary outcomes were fasting blood sugar and health-related behaviors, including physical activity and food intake, while secondary outcomes were long-term metabolic indicators, including hemoglobin A_1c_ (HbA_1c_) and BMI. All outcomes were measured by self-assessed questionnaires. No blinding was implemented for participants or care providers. However, data analysis was conducted using anonymized group labels. Eight participants were lost to follow-up. Minor missing daily entries were handled by weekly data aggregation.

**Results:**

Data from 60 participants were included in the final analysis (VR-gamified group n=16, application group n=14, counseling group n=15, and control group n=15). Linear mixed model showed significant time×group interaction effects for fasting blood sugar (*F*_15,280_=2.046; *P*=.01; partial η²=.099), physical activity (*F*_15,280.61_=2.544; *P*=.001; partial η²=.120), and food intake (*F*_15,336_*=2*.794; *P*<.001; partial η^2^=.111), indicating greater improvement over time in the VR gamification group compared with the other parallel groups. No statistically significant differences were observed for BMI or glycated hemoglobin. No serious adverse events related to the VR intervention were reported.

**Conclusions:**

VR gamified self-care interventions may potentially contribute to better management of diabetes-related symptoms and behaviors compared with conventional self-care approaches. Such gamified VR systems show promise as alternative or complementary tools for enhancing health outcomes among children and adolescents diagnosed with diabetes.

## Introduction

### Background

Self-care plays an important role in managing the progression of chronic diseases. A high level of control and effective symptom management can improve both somatic and psychological well-being in patients [1]. Self-care systems use personal goal-setting, social support, self-monitoring, and education to help patients in their daily lives [2]. Among various self-care approaches, technological methods appear to be more effective than others. The global availability and relatively low cost of technologies such as computers and smartphones make them suitable for remote monitoring and self-management [3].

Diabetes mellitus (DM) is a chronic disease that is directly influenced by lifestyle, including habits and behaviors related to glycemic control, dietary adherence, and physical activity. Therefore, encouraging motivation for self-care is a crucial aspect of diabetes treatment [4]. Poor control of diabetic symptoms can lead to mortality, while increasing physical activity and improving glycemic control can help prevent morbidity [5]. According to the “Association of Diabetes Care and Education Specialists 7” framework, any diabetes self-care approach must have a measurable impact on specific factors. These include a healthy diet, physical activity, medication management, and regular blood glucose monitoring [6].

Studies have demonstrated the relative effectiveness of health monitoring applications in enhancing daily self-care among patients diagnosed with diabetes. Numerous programs utilize health tracking processes in the context of diabetes self-care [2]. Most apps focus on blood glucose monitoring, which has been identified as the most important metric for diabetes management [6]. One well-known example is the Glucose Buddy (Azumio Inc) app, which allows users to manually log and track diabetes-related metrics such as blood glucose levels [7]. Mobile self-monitoring apps should include multiple features, such as blood glucose tracking, physical activity monitoring, food intake logging, reminders, note-taking, and social support, to be fully functional [8]. However, it has been shown that relying solely on self-monitoring features without incorporating gamification can limit the program’s effectiveness in improving self-care behaviors [9]. Platforms like SlimKicker (Henley Wing Chiu) are examples of systems designed for tracking health metrics and physical activity without incorporating engaging game design elements [10]. In contrast, MySugr (Roche diabetes care) is a diabetes management app that combines health monitoring with gamification elements to enhance user engagement and adherence [11].

Gamification is the process of using game elements to engage and motivate individuals in serious contexts unrelated to entertainment [10]. Applying gamification in health care is an effective tool for shaping patient behavior through user engagement [12]. Techniques such as self-monitoring, goal setting, reward structures, social incentives, level design, medal/badge systems, and narrative context are among the most well-known and widely used behavior change strategies [13]. Reward and incentive systems have a powerful impact on habit formation and can foster intrinsic motivation for users to repeatedly engage in desired behaviors [14].

It has been shown that gamification is more effective in establishing desirable healthy habits than health monitoring alone [9]. Evidence indicates that gamification in mobile health apps leads to measurable improvements in glycemic control and medication adherence [15]. As an example, the MySugr program allows users to track and monitor their blood glucose levels, medications, and calorie intake while using challenges, achievements, and a monster avatar to encourage regular data logging [14]. Gamification enhances user motivation and supports behavior change by making health-related tasks more enjoyable and goal-oriented [16]. Techniques like point scoring, social incentives, and predictable rewards for desired actions effectively facilitate habit formation and behavioral change by reinforcing target behaviors and shaping lasting routines [17]. One of the most notable examples is exergames, games designed to promote physical activity. Games like Bant (University Health Network) track users’ physical activity using GPS and encourage improvement [10]. It has been shown that incorporating scoring and reward systems in exergames can effectively foster sustained physical activity in users [18].

However, sole reliance on points and rewards can undermine the essence of gamification, making the experience less entertaining and engaging. Scoring elements must be integrated into a compelling and enjoyable context [14]. Studies indicate that using narrative and engaging audiovisual designs, similar to features found in video games, can convey a sense of fun, immersion, and pleasure, helping to reduce the perception of pain or discomfort [6]. A major flaw in traditional gamification designs is the absence of essential game elements such as narration, high-quality graphics, and gameplay, resulting in emotionally unappealing experiences for children and adolescents [19]. Integrating visually engaging elements and fun designs helps maintain user interest and enhances the educational value of diabetes self-care apps [20]. By using compulsion loops, cycles involving anticipation of a reward, performing an action, and receiving the reward, gamified systems can reinforce desired behavioral habits [21]. Some studies describe gamification as an art form, emphasizing that the most effective way to foster behavioral change is through well-designed reward systems. Aesthetically appealing and strategically crafted rewards engage both cognitive and motivational systems to shape lasting habits [22].

Apart from using the mentioned game design elements, creating an immersive atmosphere is necessary to reach an engaging experience. Toward this goal, the use of virtual reality (VR) platforms has been increasingly discussed in recent years [23]. Jo and Park [24] found that VR’s visual aesthetics, including shape, depth, 3D graphics, and visual design, significantly influence perceived enjoyment and adherence to the technology. VR promotes behavior change through technological novelty focused on embodied experience. By incorporating interactive designs, VR creates a sense of flow, presence, and immersion [25]. Studies show VR environments can encourage healthy behaviors, including regular exercise and proper nutrition [26].

### Objectives

The main goal of this research was to design an innovative gamification program for diabetes self-care. To our knowledge, there is limited evidence of gamified VR programs that integrate similar audiovisual and gameplay elements for diabetes self-care. This study helps address this gap by providing an initial implementation and evaluation of such a system. Following the development of the app, the next objective of our research was to assess its effectiveness in improving diabetes symptoms.

To evaluate the efficiency of every self-care method, such as our VR-gamified app, there are some metrics used in previous literature. Blood glucose monitoring is recognized as the most important metric of diabetes self-care [6]. Fasting blood sugar (FBS) is considered a short-term marker of blood glucose levels, while hemoglobin A_1c_ (HbA_1c_) serves as a long-term indicator [27]. Research has identified several key factors that influence blood glucose control, with dietary adherence and physical activity being among the most significant predictors of glycemic regulation [28]. In line with these findings, we used 5 key metrics to evaluate the effectiveness of our designed intervention. These metrics are categorized into two main groups: (1) health symptoms, including FBS, HbA_1c_, and BMI, and (2) health-related behaviors, including physical activity and food intake.

We hypothesized that experiencing our VR gamified program would have a greater impact on improving the primary self-care outcomes for diabetes. Based on this expectation, the following 5 hypotheses were formulated:

- Hypothesis 1 (H1): the improvement in the weekly average of FBS over time differs between the VR gamified group and the other experimental groups.
- Hypothesis 2 (H2): the improvement in the weekly total physical activity over time differs between the VR gamified group and the other experimental groups.
- Hypothesis 3 (H3): the improvement in the weekly average of daily food intake over time differs between the VR-gamified group and the other experimental groups.
- Hypothesis 4 (H4): the improvement in the weekly calculated BMI over time differs between the VR-gamified group and the other experimental groups.
- Hypothesis 5 (H5): the change in HbA_1c_ from pretest to posttest observations differs between the VR-gamified group and the other experimental groups.

## Methods

### Trial Design

The study used a parallel-group randomized controlled trial comprising 4 arms, with participants allocated to the groups with respective sizes of 16, 14, 15, and 15 for final analysis. Each group received one kind of self-care intervention for a 6-week period.

Two methodological changes were implemented after trial commencement. First, the physical activity measurement approach was modified from objective pedometer-based tracking to structured self-report assessment to enhance feasibility and adherence in the clinical setting. Second, the Iranian diabetes-monitoring app Idia (Mehr Nutrition Technology and Knowledge Development) was initially planned as a comparator for the VR-gamified group; however, limitations in its expected feature set necessitated its replacement with the MySugr app as one of the comparators.

### Participants

The target population for this study consisted of children and adolescents aged 7-15 years diagnosed with diabetes, living in Tehran, Iran. Inclusion criteria were (1) a formal diagnosis of diabetes, (2) aged between 7 and 15 years, (3) basic computer/VR literacy, and (4) willingness and ability to participate in a 6-week eHealth trial. Exclusion criteria included any medical conditions that would prevent safe use of VR headsets (eg, cybersickness or nausea). The research team was introduced to potential participants as specialists in health psychology affiliated with the PhD program at the University of Tehran in order to provide a clear professional context for the intervention. A total of 78 participants meeting the inclusion criteria were recruited from The Children’s Medical Center in August 2024 to November 2024, Tehran, Iran, in an offline, closed manner. Following recruitment, participants were provided with face-to-face instructions and a briefing. Written informed consent was subsequently obtained from eligible participants (n=68) who enrolled in the trial. Eight participants lost to follow-up over the 6-week period, resulting in a final sample of 60 participants (mean age 11.8, SD 1.74 years; 33, 55.0% boys and 27, 45.0% girls). All outcomes were self-assessed by online questionnaires throughout the trial timeline.

### Interventions

#### VR Gamified Group

Participants in this group engaged with the VR gamified program developed specifically for this study.

Participants engaged in one supervised VR gamified session per week for 6 consecutive weeks. Each session lasted approximately 5‐15 minutes and took place in the clinical setting. In every session, participants played one level of the KalleGhandi (Ruhollah Solat) VR game, with the difficulty adjusted based on their weekly glycemic and behavioral data obtained prior to the session. Two care providers, including one health professional and one technical assistant, were present to provide onboarding, headset setup, and technical support throughout the intervention. Participants accessed the VR game at the medical clinic following their weekly physician visit. No payment was required from participants. The game was experienced using a VR headset provided by the university, and participants did not have personal access to the device outside the clinical setting. No prompts were used, but weekly Telegram (Telegram Messenger LLP) messages were sent to participants to remind them about the intervention and related self-assessments. Apart from slight differences in the level of training, no co-intervention was provided during the trial.

**Figure 1.**
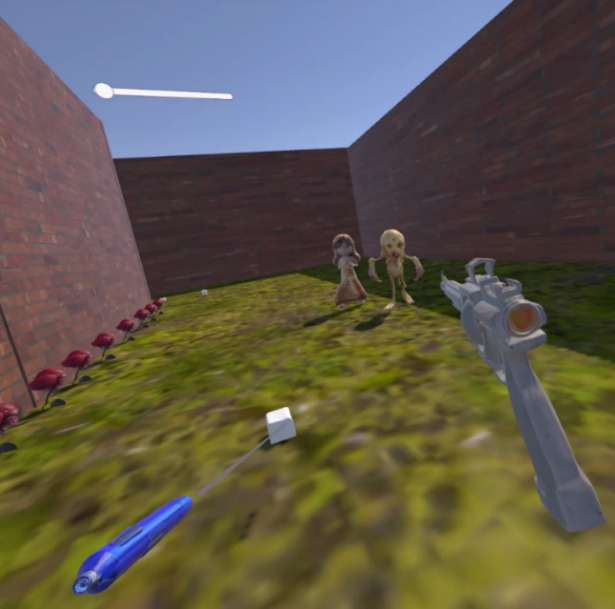
Visual overview of the “KalleGhandi” virtual reality gamification program used as an intervention in this randomized controlled trial study.

**Figure 2.**
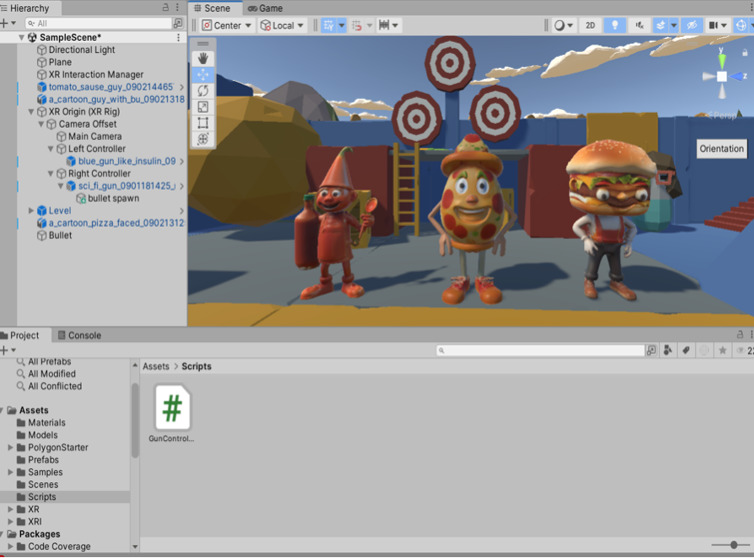
A screenshot taken from the process of making the game "KalleGhandi." Unity 2022.3.44fl game engine.

The VR game used in this study (see Figure 1) was designed and developed by the first author (RS). With part-time support from a technical game development team in Iran, the product was developed and finalized through several iterative phases between November 2023 and October 2024, using the Unity engine (version 2022.3.44f1; Unity Technologies) and tested on the Oculus Meta Quest 2 (Meta Platforms) VR headset. Figure 2 shows one screenshot of the process of making the game in the Unity engine. For ensuring quality assurance and taking feedback about the experience, the game was pilot-tested multiple times with participants without diabetes prior to the main study. The game was designed in the first-person shooter genre. In the first core mechanic of the game, players use their VR right-hand controller to shoot enemies represented as unhealthy food items such as ice cream, pizza, and cheese. The second core mechanic involves using an insulin pen with the VR left-hand controller to break down and collect sugar cubes scattered throughout the environment. Each session lasts 5 minutes, and based on the number of sugar cubes collected, participants are awarded a medal: gold, silver, or bronze.

The gamification design links each gameplay element directly to real-life diabetes management behaviors. Specifically, the target number of sugar cubes is dynamically set based on the participant’s weekly average FBS. Lower FBS averages reduce the target, making it easier to earn medals, while higher FBS averages increase the difficulty. Similarly, the player’s movement speed in the game is determined by their physical activity level, and the number of enemies (unhealthy foods) is determined by the weekly average food calories. This integration of real-life health indicators into gameplay is intended to reinforce positive self-care behaviors.

To facilitate accessibility and ensure replicability, the source codes of the KalleGhandi game are provided in Multimedia Appendix 1, while 2 versions of the game have been uploaded to the Open Science Framework (OSF):

- The Windows build (for playing on a PC with a VR headset connection) is available [29].
- The Android build (for stand-alone headset use) is available [30].

#### Usability and Tolerability Assessment

Throughout the VR intervention, participants and caregivers were monitored for usability issues, cybersickness, or discomfort. Tolerability indicators (nausea, dizziness, and visual fatigue) were recorded via caregiver observations at each session. No adverse events or dropouts due to VR intolerance occurred. All sessions were conducted under adult supervision, with optional rest breaks. In addition to observational monitoring, participants completed standardized measures to capture their subjective experience of ease of use, perceived usefulness, and overall interaction quality with the VR system. System usability was measured using the System Usability Scale developed by Brooke [31], which consists of 10 items rated on a 5-point Likert scale. Technology acceptance was measured based on the Technology Acceptance Model (TAM) construct developed by Davis [32]. The TAM model’s core constructs, perceived usefulness, and perceived ease of use were operationalized to assess users’ perception of extended reality (XR) technology. After experiencing 6 consecutive sessions, participants completed the Persian-translated versions of both questionnaires to ensure clarity and language accessibility for children and adolescent participants.

#### Application Group

Participants in this group used the commercially available diabetes self-care app MySugr (Android/iOS, standard free version available during the study period 2024‐2025) as a comparator to the VR gamified intervention.

At baseline, participants received a brief, structured training session (approximately 20‐30 min) on how to:

- Create a personal user account and log in to the app;
- Log their blood glucose measurements obtained from their home glucometer;
- Record their dietary intake and approximate calorie consumption; and
- Review the summary feedback provided by the app.

Participants used the MySugr app to log and self-monitor their blood glucose values and dietary intake. They received automated reminders prompting them to record glucose measurements and diet-related information. Participants also interacted with the app’s gamified “monster” avatar, which responded to completed logging tasks by providing visual feedback and rewards; this feature constituted the primary gamification component of the intervention.

Participants were instructed to use the app between 2 and 5 times per day, typically for several minutes per session, to enter their fasting and postprandial blood glucose values and main meals. App usage (frequency and duration) was based on self-report during follow-up visits/phone calls rather than backend log data, as we did not have access to the app’s internal usage analytics.

#### Traditional Counseling Group

This group received diabetes self-care counseling provided by professional diabetes consultancy centers in Iran.

Participants were already enrolled in diabetes care programs at either Gabric Institute or the Iranian Diabetes Center, where they routinely received counseling and self-care support alongside medication services. Counseling interactions typically occurred multiple times per week via Telegram channels and phone calls. Each participant had a primary counselor familiar with their case and family, offering tailored guidance for diabetes self-care. Institutional practices are largely uniform but not scripted.

#### Control Group

Participants in this group received medical care by an endocrinology/child-adolescent diabetes/nutrition specialist on a weekly or monthly basis but did not receive any additional psychological health-related self-care intervention.

### Outcomes

All participants (or their parents) were instructed to complete a structured data collection form throughout the 6-week study period, which is provided in Multimedia Appendix 2. The form required daily entries, including FBS, food intake, and physical activity, along with weekly entries for body weight. HbA_1c_ levels were recorded twice, once at baseline (pretest) and once after the final session (posttest). Demographic variables, including age, gender, and type of diabetes, were collected at the outset.

### Primary Outcomes

#### Weekly Average Daily Monitored FBS

Participants measured their FBS levels every morning using a personal glucometer and self-reported the values. The recorded daily FBS values were then used by the experimenters to calculate a weekly average for each participant.

FBS values were measured by participants using personal home glucometers commonly available in community pharmacies. Because measurements were conducted in real-world settings, different commercially available glucometer brands like Accu-Check were used. Participants were instructed to measure blood glucose in the morning after an overnight fast (at least 8 h) following standard self-monitoring procedures recommended for home glucose testing. No continuous glucose monitoring devices were used in this study. Daily FBS values were recorded by participants and submitted through the study reporting forms.

#### Weekly Sum of Daily Calculated Physical Activity

Physical activity was quantified using a bar metric; one bar equaled either 30 minutes of vigorous activity (eg, gym workouts or swimming) or 60 minutes of moderate activity (eg, jogging). The weekly sum was calculated based on this definition.

As the study prioritized pragmatic feasibility in real-world settings, we intentionally relied on structured self-report measures rather than resource-intensive objective tools such as pedometers to record daily physical activity. Participants and parents received brief standardized training on reporting physical activity using the bar metric with examples of common daily exercises. This change in measurement methods happened shortly after the commencement of the trial.

#### Weekly Average Daily Calculated Food Intake

Participants either reported their total daily caloric intake directly or reported their food descriptions, from which caloric values were estimated. A weekly average of daily caloric intake was then computed.

To preserve pragmatic feasibility and participants’ adherence, dietary intake was assessed using a self-assessment approach, rather than resource-intensive objective methods. Participants and parents received brief standardized training on estimating daily caloric intake using a nationally recognized Iranian food-calorie database (Dr Kermani’s online nutrition resource [33]). Although self-report measures are inherently vulnerable to reporting bias, self-assessment contributed to operational feasibility, participant adherence, and real-world generalizability of the findings.

### Secondary Outcomes

#### Weekly Calculated BMI

Participants self-reported their weight on a weekly basis. BMI was calculated using the standard formula: weight (kg) divided by height squared (m²), which had been reported at first.

#### HbA_1c_ Pretest and Posttest

HbA_1c_ levels were measured twice via laboratory blood tests, once before the intervention (pretest) and once at the conclusion of the study (posttest), serving as a long-term indicator of blood glucose control.

Participants were instructed to undergo HbA_1c_ testing at a certified clinical laboratory of their choice using standard methods routinely used in clinical practice (eg, high-performance liquid chromatography or equivalent). All laboratories were accredited according to national standards. As HbA_1c_ measurements were collected from multiple laboratories, the same laboratory was not used for all participants; however, HbA_1c_ is a standardized biomarker with minimal interlaboratory variability.

### Sample Size

Sample size was calculated using G*Power (Heinrich Heine University Düsseldorf) software based on a repeated-measures ANOVA design with 4 groups. Assuming a medium effect size (*f*=.25), a significance level of α=.05, and statistical power of 0.80, the minimum required total sample size was estimated to be 52 participants. To account for potential attrition, additional participants were recruited. No interim analyses or stopping guidelines were defined or conducted.

### Randomization

Participants were randomly assigned to one of the 4 groups using computer-generated stratified block randomization based on age and sex. The allocation sequence was generated by the technical assistant who was not involved in the study assessments. Allocation concealment was maintained through a sequentially numbered process, and the care provider responsible for briefing and tutorials remained unaware of group assignments until the moment of allocation.

### Blinding

Due to the nature of the interventions, neither participants nor care providers could be blinded to group assignments. All outcome data were self-reported via online questionnaires, with no external outcome assessor involved. However, the data analyst was provided with anonymized group labels to minimize potential analysis bias. To reduce preconceived biases about the intervention of interest and comparators**,** the informed consent focused on the shared goal of diabetes improvement and participants’ compensation, ensuring a neutral presentation of all study arms. No similarity of a placebo intervention was applicable here.

### Statistical Methods

Given the repeated measurements of outcomes, a linear mixed model (LMM) was used. To investigate significant differences between groups, one-way ANOVA and paired-samples 2-tailed *t* tests were used. A correlation analysis was also used as an additional analysis. All statistical analyses were performed using SPSS Statistics (version 27; IBM Corp). The significance level was set at α=.05 for all statistical tests.

Handling of missing data: the variables BMI and HbA_1c_ contained no missing data, as BMI was derived from weekly weight measurements and HbA_1c_ was collected only at the pretest and posttest time points. The other 3 outcomes were constructed at the weekly level, as it is an established, accepted practice in mobile health and diabetes care studies. For FBS, physical activity, and food intake**,** missing values existed only in the daily records. According to the operational definitions of the study, these variables were aggregated to the weekly level using the mean or sum of the available daily entries. Weeks with partial daily logs were still computable, because weekly values can be calculated based on the days that were present rather than requiring a complete daily set. As a result of this aggregation process, the final analytic dataset contained no missing values for any of the weekly variables. No imputation procedure was used.

### Reporting Guideline

The study was reported in accordance with the CONSORT (Consolidated Standards of Reporting Trials) 2010 statement. The preparation of the manuscript followed the CONSORT-EHEALTH (Consolidated Standards of Reporting Trials of Electronic and Mobile Health Applications and Online Telehealth) reporting framework (Checklist 1) [34], and the abstract was structured according to the CONSORT guidance for abstracts [35], to ensure transparent and standardized reporting.

### Ethical Considerations

Ethical approval was obtained from the ethical committee of Tehran University’s psychology department, under the supervision of the Ministry of Health and Medical Education (MOHME), indexed as IR.UT.PSYEDU.REC.1404.106. All procedures were conducted in accordance with the ethical standards of the institutional research committee and the Declaration of Helsinki. Prior to participation, offline written informed consent was obtained from all participants or their parents. Participants were informed about the purpose of the study, trial procedures, and their right to withdraw at any time without any consequences. Participant privacy and confidentiality were strictly protected throughout the study. All collected data were deidentified and stored using coded identifiers, and no personally identifiable information was included in the analysis or reporting of the results. Access to the data was restricted to the research team and maintained in secure files in accordance with institutional ethical guidelines. No identification of individual participants in any image of the manuscript or supplementary material is possible. Participants received a small compensation to acknowledge the time and effort required to take part in the study. The type and amount of compensation were determined in accordance with the institutional ethical guidelines and were provided equally to all participants regardless of group allocation or study outcomes. No personal or health-related information was collected or transmitted through the VR application. In addition, XR safety procedures were implemented, including supervised use and monitoring for symptoms such as cybersickness, nausea, or any other bodily discomfort.

## Results

### Participant Flow

**Figure 3.**
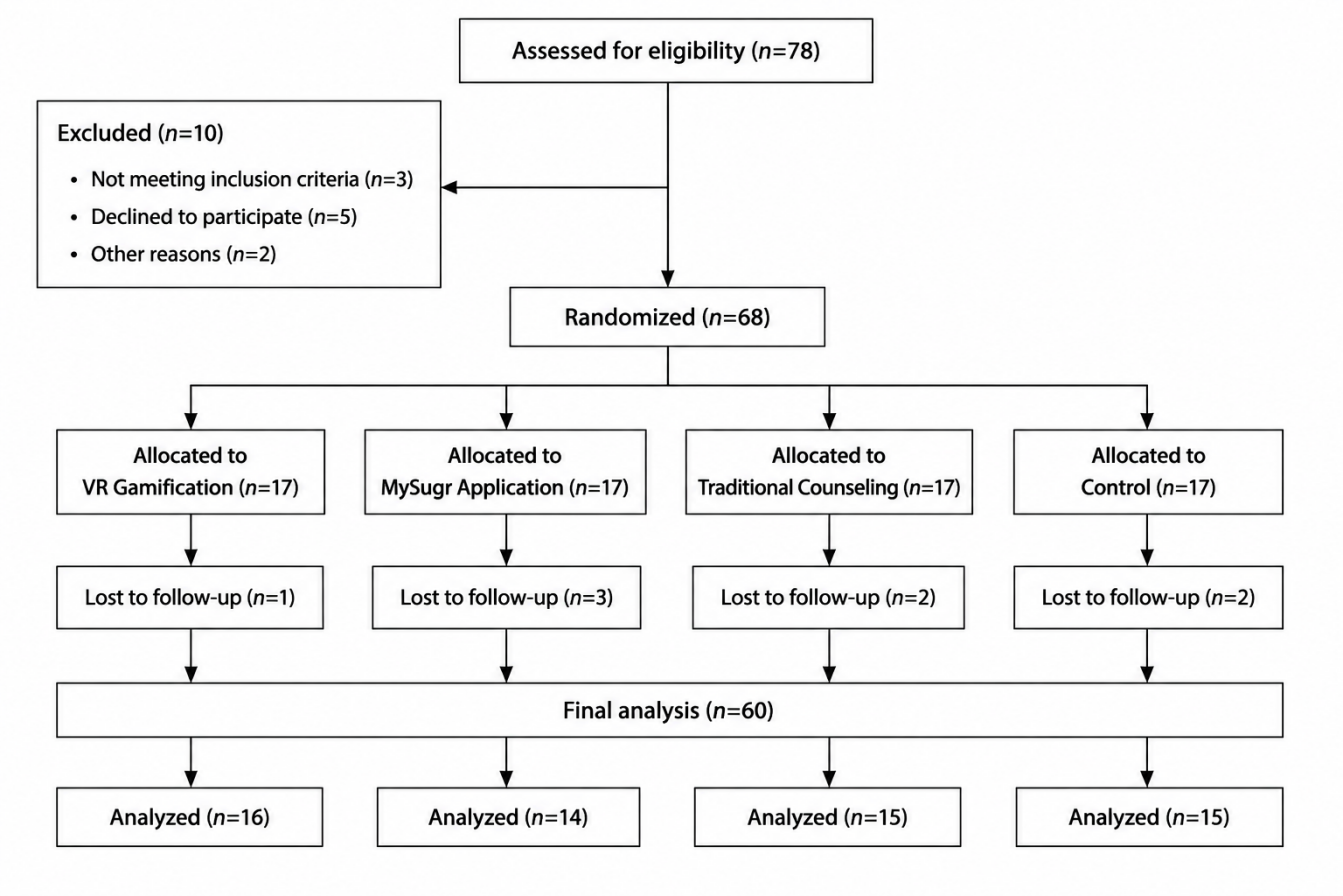
Participant flow diagram of randomized controlled trial (RCT) program: “Improving diabetes health symptoms by using a specialized VR gamification self-care method: A parallel-group randomized controlled trial” according to CONSORT (Consolidated Standards of Reporting Trials) 2025. Eligibility assessment, enrollment, random allocation, follow-up status, and number of participants analyzed in each group. The study recruited children and adolescents diagnosed with diabetes in Tehran, Iran, from August 2024 to November 2024 and was conducted in December 2024-February 2025.

A total of 78 individuals were recruited and assessed for eligibility. After the initial briefing**,** 10 were excluded (3 did not fully meet the inclusion criteria, 5 declined to participate, and 2 for other reasons). A total of 68 participants were randomized using stratified block randomization into 4 parallel groups with equal-sized arms (n=17). During the 6-week study period, a total of 8 participants were lost to follow-up. Consequently, 60 participants were included in the final analysis. The detailed participant flow is illustrated in Figure 3.

### Recruitment

Participants were recruited and enrolled between August and November 2024. The intervention phase of the trial was conducted from December 2024 to February 2025, during which each participant completed a 6-week follow-up period. The trial concluded as planned without early termination.

### Baseline Data

Table 1 presents the baseline demographic and clinical characteristics of participants in the 4 study groups prior to the intervention.

**Table 1.** Baseline characteristics of participants in each group. Baseline comparability can be interpreted. Gender is coded as 1=male and 2=female.

Characteristic	VR gamification (n=16)	MySugr app (n=14)	Traditional counseling (n=15)	Control (n=15)
Age (years)				
Mean (SD)	11.75 (2.05)	11.86 (1.66)	11.80 (1.74)	11.60 (1.84)
Range	7‐15	9‐15	9‐15	8‐15
Gender, mean (SD)	1.44 (0.51)	1.43 (0.51)	1.47 (0.52)	1.47 (0.52)
Diabetes type, mean (SD)	1.25 (0.44)	1.28 (0.46)	1.26 (0.45)	1.26 (0.45)
BMI (kg/m²)				
Mean (SD)	23.85 (5.55)	24.26 (6.62)	24.47 (6.74)	23.94 (6.18)
Range	18.3‐36.2	16.2‐36.2	16.5‐35.3	18.8‐37.1
FBS^a^ (mg/dL)				
Mean (SD)	123.63 (18.95)	121.14 (18.19)	119.27 (20.22)	119.33 (18.23)
Range	93‐163	83‐155	81‐158	84‐152
HbA_1c_^b^ (%)				
Mean (SD)	7.94 (0.79)	7.66 (0.89)	8.13 (1.10)	7.51 (0.77)
Range	6.5‐9.4	6.7‐9.6	6.5‐10.2	6.2‐8.6

a FBS: fasting blood sugar.

b HbA_1c_: glycated hemoglobin.

### Numbers Analyzed

Data from 60 participants with complete outcome data were included in the final analysis. The denominator for all analyses corresponds to participants who completed the study and provided postintervention measurements. Analyses were conducted according to the originally assigned groups. The analyzed sample sizes were 16 in the VR gamified group, 14 in the MySugr app group, 15 in the traditional counseling group, and 15 in the control group.

### Outcomes

The results of the LMM for all 5 outcomes are summarized in Table 2.

**Table 2.** Linear mixed model (LMM) results for trial outcomes. Model fit indices the Akaike information criterion (AIC), residual variance, and fixed-effects tests for time, condition, and their interaction are reported for all outcomes (fasting blood sugar [FBS], physical activity, food intake, BMI, and hemoglobin A_1c_ [HbA_1c_]) in the parallel-group randomized controlled trial (RCT) evaluating a specialized virtual reality (VR)–gamification self-care program for children and adolescents diagnosed with diabetes. *P* values and effect sizes are reported, respectively.

Outcome and source	*F* (df)	*P* value	Partial η^2^	Model fit (AIC)	Residual variance
FBS				2383.29	29.05 (2.45)
Time	0.269 (5,280)	.93	.005		
Time×condition	2.046 (15,280)	.01	.099		
Condition	0.171 (3,39.91)	.91	.001		
Physical activity				1089.44	0.97 (0.08)
Time	1.580 (5,280.61)	.16	.027		
Time×condition	2.544 (15,280.61)	.003	.120		
Condition	14.399 (3.55.96)	<.001	.430		
Food intake				4138.80	4737.43 (400.39)
Time	3.487 (5,336)	.004	.059		
Time×condition	2.794 (15,336)	<.001	.111		
Condition	0.115 (3.336)	.95	.001		
BMI				429.61	0.03 (0.01)
Time	0.602 (5,280)	.69	.011		
Time×condition	0.660 (6.876,128.318)	.70	—		
Condition	0.021 (3,40.19)	.99	.034		
HbA_1c_				306.57	0.71 (0.09)
Time	0.23 (1,112)	.63	.002		
Time×condition	0.064 (3,112)	.97	.002		
Condition	2.980 (3,112)	.03	.074		

### FBS

**Figure 4.**
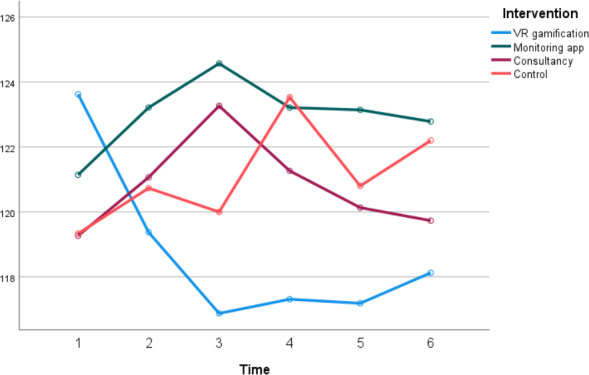
Changes in fasting blood sugar (FBS) over time across intervention conditions. Trajectories show mean FBS values at 6 measurement points for the VR-Gamification, MySugr app, traditional counseling, and control group in the present randomized controlled trial study. Population: children and adolescents diagnosed with diabetes (age: 7‐15 years), Tehran, Iran. Conducted in December 2024 to February 2025. VR: virtual reality.

An LMM was fitted with time, condition**,** and their interaction as fixed effects and a random intercept with autoregressive correlation of order 1 (AR1) covariance for participants. Model fit indices indicated adequate performance (Akaike information criterion [AIC]=2383.29), suggesting that adding time and the interaction improved overall model parsimony relative to the reduced model. The results of the LMM, as reported in Table 2, indicate that the time×condition interaction effect was statistically significant (*F*_15,280_= 2.046; *P*=.01; partial η^2^≈.099). However, no significant main effects were observed for time (*F*_5,280_*=*0.269*; P*=.93; partial η^2^≈.005) or condition (*F*_3,39.91_=0.171; *P*=.91; partial η^2^=.001), suggesting that overall changes over time and between-group differences were not statistically significant in isolation. Random-effects estimates indicated substantial between-subject variability (random-intercept variance=143.45, SE 29.92). The AR1 correlation parameter was negative (ρ=−.19), indicating weak inverse correlation between adjacent repeated measurements. Residual variance was 29.05 (SE 2.45). The temporal changes in the weekly average of FBS across the 4 experimental groups over 6 repeated measurements are illustrated in Figure 4. The significant interaction effect indicates that the pattern of FBS improvement over time varied by group. Specifically, participants in the VR gamified group showed a distinct trend in reducing FBS compared to other groups, with estimated marginal means decreasing from 123.625 mg/dL, 95% CI 114.147-133.103 at baseline to 118.125 mg/dL, 95% CI 110.387-125.863 at week 6. A paired-samples *t* test was conducted to examine the change in average FBS levels within the VR gamified group between the first and last sessions. The results revealed a significant decrease in FBS levels over time (*t*_15_=−2.612; *P*=.02; mean difference=−5.500, 95% CI −1.012 to −9.987; Cohen *d*=0.653, 95% CI 0.102-1.186), indicating improved glycemic control among participants in this group, which is in line with recent studies [36]. This finding supports hypothesis 1.

### Physical Activity

**Figure 5.**
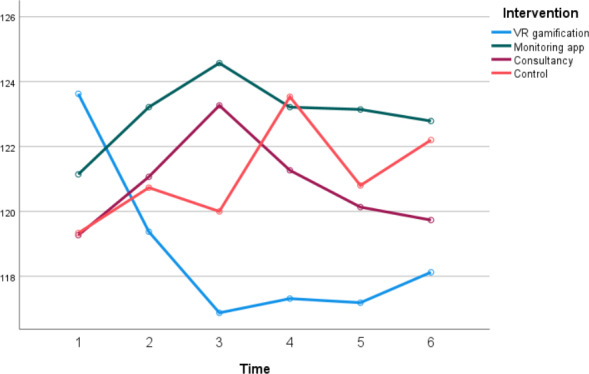
Changes in physical activity (calculated as weekly sum of daily physical activity) over time across intervention conditions. One bar equaled either 30 minutes of vigorous activity (eg, gym workouts or swimming) or 60 minutes of moderate activity (eg, jogging). Trajectories show mean physical activity values at 6 measurement points for the VR-gamification, MySugr app, traditional counseling, and control group in the present randomized controlled trial study. Population: children and adolescents diagnosed with diabetes (age: 7‐15 years), Tehran, Iran. Conducted in December 2024-February 2025. VR: virtual reality.

An LMM was estimated with time**,** condition, and their interaction as fixed effects and a random intercept with an AR1 covariance structure for participants. Model fit indices (AIC=1089.44) indicated a relatively well-fitting model with adequate parsimony for the repeated-measures structure. According to Table 2, LMM results revealed a significant main effect of condition (*F*_3,55.96_=14.399; *P*<.001; partial η^2^≈.435) and a nonsignificant main effect of time (*F*_5,280.61_=1.580; *P*=.16; partial η^2^≈.027). The interaction effect of time×condition was significant (*F*_15,280.61_= 2.544; *P*=.001; partial η^2^≈.120), indicating that changes in physical activity over time differed significantly between the VR gamified group and the other experimental groups. Random-effects estimates showed modest between-subject variability (random-intercept variance=0.155; SE 0.0678). The AR1 correlation parameter was positive (ρ=0.656), indicating moderate positive correlation between adjacent repeated measurements. Residual variance was 0.979 (SE 0.083). Figure 5 illustrates the temporal changes in weekly physical activity across the 4 experimental groups. While physical activity levels remained relatively stable in the control, traditional, and app-based groups, the VR gamified group exhibited a noticeable upward trend over time. Thus, hypothesis 2 is supported.

The one-way ANOVA analysis for physical activity during the last session revealed a significant difference in average physical activity between the VR gamified group and the other experimental groups (*F*_3,56_=21.234; *P*<.001; partial η^2^=.532). A paired-samples *t* test result showed improvement of physical activity in VR group between the first and last sessions (*t*_15_=3.850; *P*=.002; mean difference=1.625, 95% CI 0.725-2.524; Cohen *d*=0.963, 95% CI 0.355-1.549). These findings align with recent meta-analytic evidence demonstrating a strong positive effect of exergames on physical activity levels [37].

### Food Intake

**Figure 6.**
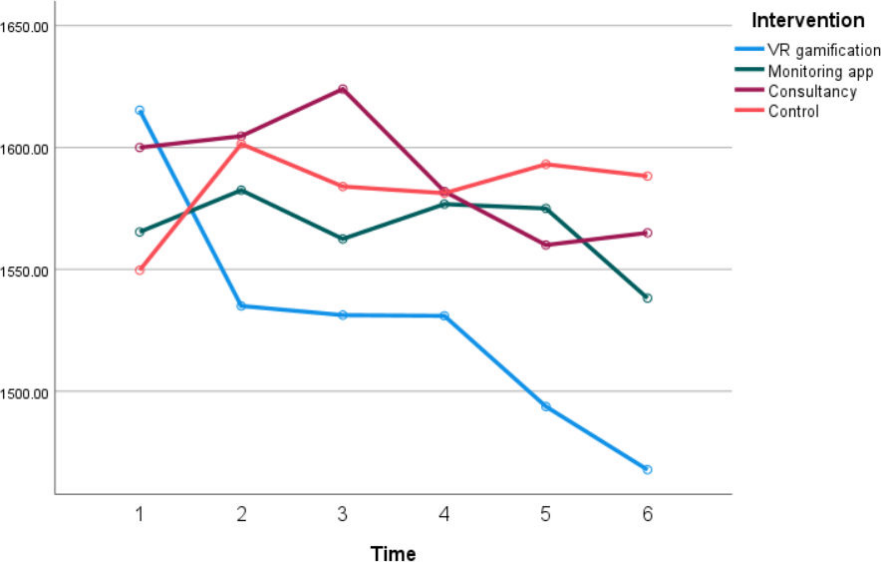
Changes in food intake (calculated as weekly average of daily calories) over time across intervention conditions. Trajectories show mean daily calorie values at 6 measurement points for the virtual reality (VR)–gamification, MySugr app, traditional counseling, and control group in the present randomized controlled trial study. Population: children and adolescents diagnosed with diabetes (age: 7‐15 years), Tehran, Iran. Conducted in December 2024-February 2025. VR: virtual reality.

An LMM was estimated with time**,** condition, and their interaction as fixed effects and a random intercept with an AR1 covariance structure for participants. Model-fit indices (AIC=4138.80) indicated acceptable fit for the repeated-measures design. According to the LMM results presented in Table 2, the main effect of condition was not significant (*F*_3,336_*=*0.115; *P*=.95, partial η^2^≈.001), while the main effect of time was significant (*F*_5,336_*=*3.487; *P*=.004; partial η^2^≈.059), indicating that the observed downward trend over time was unlikely due to chance. The time×condition interaction effect was significant (*F*_15,336_=2.794; *P*<.001; partial η^2^≈.111), suggesting that the pattern of change in food intake over time differed between the VR gamified group and the other groups. Random-effects estimates showed large between-subject variability (random-intercept variance=24,595.20,.20; SE 4685.39). The AR1 correlation parameter was reported as ρ=1.000; however, SPSS notes this value is redundant, typically indicating boundary estimation or insufficient information for precise AR1 correlation. Residual variance was 4737.43 (SE 400.39). Figure 6 illustrates the temporal changes in the weekly average calculated food intake across the 4 experimental groups. While food intake fluctuated irregularly in the control, traditional, and app-based groups, the VR gamified group showed a consistent downward trend. Thus, hypothesis 3 is supported.

Results of the paired-samples *t* test for the food intake variable in the VR gamified group showed a significant decrease in average food intake from the first to the last session (*t*_15_=−3.850; *P*=.002; mean difference=−147.500, 95% CI −0.725 to –2.524; Cohen *d*=0.963, 95% CI 0.355-1.549), which is in line with recent studies [38].

### BMI

**Figure 7.**
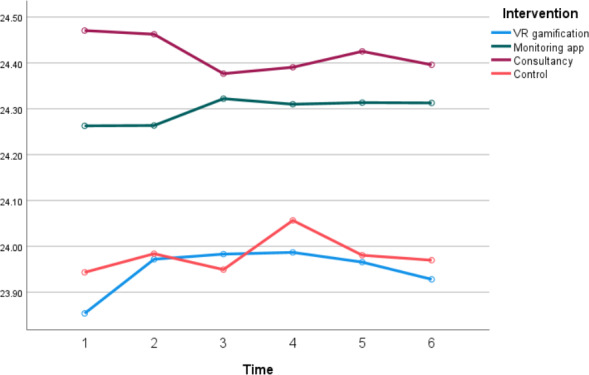
Changes in BMI over time across intervention conditions. Trajectories show BMI mean values at 6 measurement points for the virtual reality (VR)–gamification, MySugr app, traditional counseling, and control group in the present randomized controlled trial study. Population: children and adolescents diagnosed with diabetes (age: 7‐15 years), Tehran, Iran. Conducted in December 2024-February 2025. VR: virtual reality.

An LMM was fitted with time, condition**,** and their interaction as fixed effects and a random intercept with an AR1 covariance structure for participants. Model-fit indices (AIC=429.61) indicated that the model adequately captured the repeated-measures structure. According to Table 2, the LMM results for BMI showed no significant effects. For time effect (*F*_5,280_=0.602; *P*=.69; partial η^2^≈.011), for condition effect (*F*_3,40.19_=0.021; *P*=.99; partial η^2^≈.000), and for the time×condition interaction effect (*F*_15,280_=0.660; *P*=.82; partial η^2^=.034). Random-effects estimates indicated substantial between-subject variability (random-intercept variance=21.17; SE 4.32). The AR1 correlation parameter was slightly negative (ρ=–0.209), indicating weak inverse correlation between adjacent repeated BMI measurements. Residual variance was 0.0399 (SE 0.00337). Figure 7 illustrates that the weekly calculated BMI remains relatively stable and consistent across all 4 experimental groups throughout the study period. This suggests that the interventions did not have a significant impact on BMI changes over time. Therefore, hypothesis 4 is rejected.

### HbA_1c_

**Figure 8.**
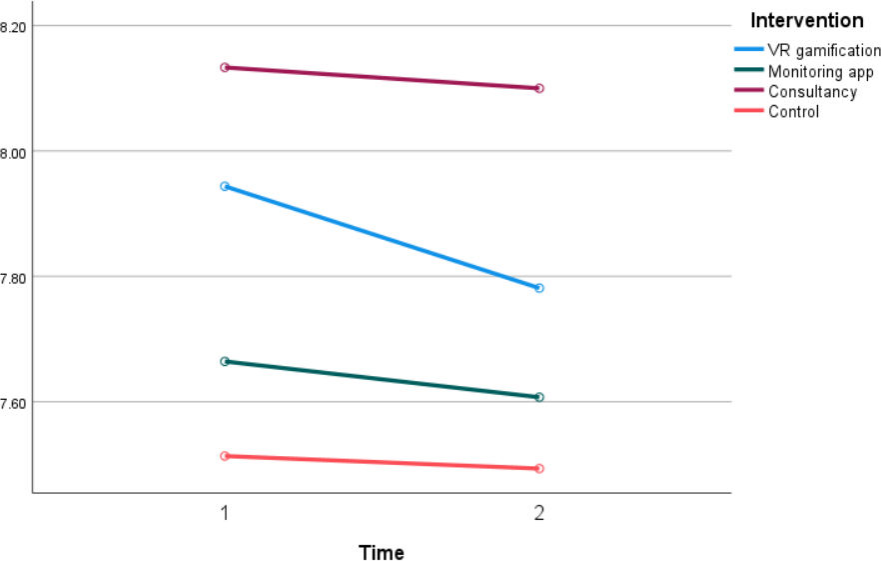
Changes in glycated hemoglobin (HbA_1c_) over time across intervention conditions. Trajectories show mean HbA_1c_ values at 2 measurement points: pretest and posttest for the virtual reality (VR)–gamification, MySugr app, traditional counseling, and control group in the present randomized controlled trial study. Population: children and adolescents diagnosed with diabetes (age: 7‐15 years), Tehran, Iran. Conducted in December 2024-February 2025. VR: virtual reality.

An LMM was fitted with condition, time, and their interaction as fixed effects. A random intercept for participants was included, and the identity covariance structure was used. The model showed acceptable fit (AIC=306.57). The LMM results presented in Table 2 reveal significant effect for the condition effect (*F*_3,112_=2.980; *P*=.03; partial η^2^≈.074), the time effect (*F*_1,112_=0.230; *P*=.63; partial η^2^≈.002), and the time×condition interaction effect (*F*_3,112_= 0.064; *P*=.97; partial η^2^=0.002). Although the main effect of condition reached statistical significance in the mixed model, this effect reflects baseline differences between groups rather than any intervention-related change**,** as the related effect size is small (partial η^2^≈.074). Therefore, no meaningful or clinically relevant effect of the intervention on HbA_1c_ can be inferred. As Figure 8 shows, the pattern of change between the 2 time points did not differ across conditions. Therefore, hypothesis 5 is rejected. Random intercept variance was very small (0.00098), reflecting minimal between-subject variability after accounting for fixed effects. Residual variance was 0.719 (SE 0.096).

Despite the strong correlation between HbA_1c_ and FBS (*r*_58_=0.641; *P*<.001; 95% CI 0.469-0.770), our results showed no significant change in HbA_1c_ levels between the pretest and posttest. This finding contrasts with previous studies (eg, Kerfoot et al [39]), which reported significant changes in HbA_1c_ levels over a 12-month period (*P*=.048). The discrepancy may be attributed to the relatively short duration of the current intervention.

### Ancillary Analysis

One exploratory analysis was performed, consisting of a correlation analysis to examine associations between behavioral and metabolic outcomes. As Table 3 shows, significant associations were observed between changes in FBS and physical activity (r_58_=–0.422, 95% CI –0.610 to –0.188; *P*<.001) and between changes in physical activity and food intake (r_58_= –0.292, 95% CI –0.509– to 0.041; *P*=.02), indicating linked behavioral and metabolic shifts. This pattern aligns with prior findings on the relationship between physical activity and blood glucose levels [40].

**Table 3.** Pearson correlations among change scores for trial outcomes. Correlation coefficients show the associations between changes in fasting blood sugar (FBS), physical activity, and food intake across 60 participants of the present parallel group randomized controlled trial study. Population: children and adolescents diagnosed with diabetes (age: 7‐15 years), Tehran, Iran. Conducted in December 2024-February 2025.

Variable	Changes in FBS^a^	Changes in physical activity	Changes in food intake	
Changes in FBS	1	–0.422	0.082	
Changes in physical activity	–0.422	1	–0.292	
Changes in food intake	0.082	–0.292	1	

a FBS: fasting blood sugar.

### Harms

All XR sessions were completed without any adverse events or early terminations. No instances of cybersickness, visual discomfort, dizziness, or excessive fatigue were reported, and no participant required a session to be stopped prematurely. Adherence to the intervention was exceptionally high, with all children attending and completing all scheduled sessions. Participants in the VR group (n=16) demonstrated high perceived usability, with a mean System Usability Scale score of 74.2 (SD 7.9). According to established usability benchmarks, this value falls within the Excellent range, indicating that the VR environment was experienced as intuitive, consistent, and easy to navigate. No individual item showed unusually low ratings, suggesting that participants were able to interact with the system with minimal cognitive effort or technical difficulty. TAM responses further supported strong user acceptance of the VR system. Perceived usefulness yielded a mean score of 5.3/7 (SD 0.7)**,** while perceived ease of use was rated 5.6/7 (SD 0.6). These values align with a moderately good acceptance profile, reflecting participants’ perceptions that the VR platform was beneficial, motivational, and easy to learn.

## Discussion

### Principal Findings

The present study aimed to evaluate the effectiveness of a VR gamified program in improving metabolic indicators and health-related behaviors among children and adolescents diagnosed with diabetes. The intervention was compared with 3 comparators, a gamified mobile app (MySugr), traditional consultation, and standard medical care as a control group, in a longitudinal 6-week parallel-groups RCT design. Overall, the results indicate that the VR gamified intervention produced the most consistent improvements in behavioral outcomes, including physical activity and dietary control, compared to the other 3 parallel groups. Moderate yet promising improvements in FBS were also observed in the VR gamified group relative to the other intervention groups, suggesting that the VR gamified method can potentially yield an improved level of short-term glycemic control compared to other methods. In contrast, the intervention did not produce significant improvements in long-term metabolic indicators, including BMI or HbA_1c_, in the VR gamified group compared to other interventions.

The behavioral outcomes including physical activity and dietary control are consistent with recent studies, indicating that interactive gamified environments can enhance the development of sustained health behaviors [41]. The significant improvement in short-term glycemic control (FBS) alongside a lack of significant improvements in long-term metabolic control markers, including HbA_1c_ and BMI, are in line with recent studies, as long-term metabolic indicators typically require sustained behavioral modification over longer periods to demonstrate measurable changes [42]. The overall improvement trends observed in the VR-gamified group highlight the importance of emotional engagement in self-care interventions. This finding is consistent with previous studies emphasizing that interactive gameplay and immersive audio-visual experiences are critical factors in the success of health gamification systems [43]. In contrast to conventional health-tracking programs that rely primarily on point/badge reward structures, the VR gamified intervention used in this study integrated immersive 3D environments with action-based gameplay that directly represented users’ health symptoms and behaviors. Mentioned features may explain the stronger behavioral engagement observed in the VR gamified group and may also clarify why traditional point/badge gamification approaches may produce weaker effects, as reported in some studies [44].

### Applications and Limitations

Real-world implementation of this VR intervention could align with existing pediatric diabetes education pathways in which families and educators work together to support self-care. In practice, parents can help supervise home-based sessions, while school health staff may provide complementary reinforcement during the day. Follow-up by diabetes educators or community nurses could be incorporated into routine clinical visits or remote check-ins, enabling the VR experience to serve as an adjunct to ongoing education rather than an isolated tool [45]. However, equitable access to VR remains a challenge. Variability in the availability of VR devices and limited technological resources in some families or schools may restrict adoption. To mitigate such barriers, future implementations may require shared devices in clinical settings or mobile-based alternatives to ensure broader accessibility. Due to the experimental design of this study, the external validity and generalizability of the findings should be interpreted with caution.

Several limitations should be considered when interpreting the findings of this study. First, the relatively small sample size (n=60) may limit the generalizability of the results. Second, the intervention period was relatively short (6 weeks), which may not have been sufficient to observe meaningful changes in long-term metabolic indicators, including BMI and HbA_1c_. Third, outcomes were measured through self-reported data, which may have caused reporting bias. Future research should therefore incorporate larger samples, longer intervention durations, and objective behavioral tracking tools to provide more robust evidence regarding the effectiveness of VR gamified self-care interventions.

The VR gamified experience developed in this study appears to offer a promising complement or alternative to existing self-care methods, with preliminary indications of potentially enhanced engagement and outcomes. However, interpretations of the behavioral findings should be calibrated to the limitations of measurements, including reporting bias, limited sample size, short duration of the intervention, variations in adherence and fidelity, and the novelty effect of the VR game. Results should be understood as preliminary behavioral signals rather than evidence of clinical improvements. While the program effectively fostered behavioral engagement and adherence trends, no clinically causal inference can be drawn. Future studies with new game mechanics, more engaging designs, larger samples, longer follow-ups, and objective tracking tools can scale the usability of the present intervention and extend related findings.

### Conclusion

This study combined gamification elements with immersive VR features to develop a novel self-care system for children and adolescents diagnosed with diabetes. The intervention was grounded in the behavioral economics framework, specifically the theoretical sequence of reward, motivation, behavior, and habit formation, which is central to modern gamification approaches. By offering engaging gameplay within an immersive VR environment, the game established a direct connection between real-life diabetes and in-game mechanics. The goal was to foster intrinsic motivation through in-game rewards, thereby encouraging users to sustain desirable self-care behaviors. Findings support the potential effectiveness of VR gamified intervention, highlighting its power to serve as a complementary or alternative digital tool for enhancing diabetes self-management. However, clinical implications should be interpreted cautiously.

## Supplementary material

10.2196/81402 Multimedia Appendix 1 The game source codes.

10.2196/81402 Multimedia Appendix 2 Diabetes markers data collection form.

10.2196/81402 Checklist 1 CONSORT-eHEALTH checklist (V 1.6.1).

## Abbreviations

AIC: Akaike information criterion
AR1: autoregressive correlation of order 1
CONSORT: Consolidated Standards of Reporting Trials
CONSORT-EHEALTH: Consolidated Standards of Reporting Trials of Electronic and Mobile Health Applications and Online Telehealth
DM: diabetes mellitus
FBS: fasting blood sugar
HbA_1c_: glycated hemoglobin
LMM: linear mixed model
MOHME: Ministry of Health and Medical Education
OSF: Open Science Framework
TAM: Technology Acceptance Model
VR: virtual reality
XR: extended reality
