# Supplementary material for: Improving Health-Related Symptoms and Behaviors in Children and Adolescents Diagnosed With Diabetes by Using a Virtual Reality–Gamified Self-Care Method: Randomized Controlled Trial
Source: JMIR XR Spat Comput. 2026 Jun 10;3:e81402. doi: 10.2196/81402 (PMC13252703; doi:10.2196/81402)
Supplement: Multimedia Appendix 2 [file xr-v3-e81402-s002.docx]

ID Number:

Age:

Gender:

Type of Diabetes:

Height (cm):

Date of the first recording day:

**FBS:**

| **Day 7=** | **Day 6=** | **Day 5=** | **Day 4=** | **Day 3=** | **Day 2=** | **Day 1=** | **Week 1** |
| --- | --- | --- | --- | --- | --- | --- | --- |
| **Day 7=** | **Day 6=** | **Day 5=** | **Day 4=** | **Day 3=** | **Day 2=** | **Day 1=** | **Week 2** |
| **Day 7=** | **Day 6=** | **Day 5=** | **Day 4=** | **Day 3=** | **Day 2=** | **Day 1=** | **Week 3** |
| **Day 7=** | **Day 6=** | **Day 5=** | **Day 4=** | **Day 3=** | **Day 2=** | **Day 1=** | **Week 4** |
| **Day 7=** | **Day 6=** | **Day 5=** | **Day 4=** | **Day 3=** | **Day 2=** | **Day 1=** | **Week 5** |
| **Day 7=** | **Day 6=** | **Day 5=** | **Day 4=** | **Day 3=** | **Day 2=** | **Day 1=** | **Week 6** |

**Physical Activity (Bars):**

| **Day 7=** | **Day 6=** | **Day 5=** | **Day 4=** | **Day 3=** | **Day 2=** | **Day 1=** | **Week 1** |
| --- | --- | --- | --- | --- | --- | --- | --- |
| **Day 7=** | **Day 6=** | **Day 5=** | **Day 4=** | **Day 3=** | **Day 2=** | **Day 1=** | **Week 2** |
| **Day 7=** | **Day 6=** | **Day 5=** | **Day 4=** | **Day 3=** | **Day 2=** | **Day 1=** | **Week 3** |
| **Day 7=** | **Day 6=** | **Day 5=** | **Day 4=** | **Day 3=** | **Day 2=** | **Day 1=** | **Week 4** |
| **Day 7=** | **Day 6=** | **Day 5=** | **Day 4=** | **Day 3=** | **Day 2=** | **Day 1=** | **Week 5** |
| **Day 7=** | **Day 6=** | **Day 5=** | **Day 4=** | **Day 3=** | **Day 2=** | **Day 1=** | **Week 6** |

**Food Intake (Calories):**

| **Day 7=** | **Day 6=** | **Day 5=** | **Day 4=** | **Day 3=** | **Day 2=** | **Day 1=** | **Week 1** |
| --- | --- | --- | --- | --- | --- | --- | --- |
| **Day 7=** | **Day 6=** | **Day 5=** | **Day 4=** | **Day 3=** | **Day 2=** | **Day 1=** | **Week 2** |
| **Day 7=** | **Day 6=** | **Day 5=** | **Day 4=** | **Day 3=** | **Day 2=** | **Day 1=** | **Week 3** |
| **Day 7=** | **Day 6=** | **Day 5=** | **Day 4=** | **Day 3=** | **Day 2=** | **Day 1=** | **Week 4** |
| **Day 7=** | **Day 6=** | **Day 5=** | **Day 4=** | **Day 3=** | **Day 2=** | **Day 1=** | **Week 5** |
| **Day 7=** | **Day 6=** | **Day 5=** | **Day 4=** | **Day 3=** | **Day 2=** | **Day 1=** | **Week 6** |

**Weight (Kg):**

|  | **Week 1** |
| --- | --- |
|  | **Week 2** |
|  | **Week 3** |
|  | **Week 4** |
|  | **Week 5** |
|  | **Week 6** |

**HbA1c:**

| **Post-test** | **Pre-test** |
| --- | --- |
